# Genetic Diversity and Distribution of Virulence-Associated Genes in *Y. enterocolitica* and *Y. enterocolitica*-Like Isolates from Humans and Animals in Poland

**DOI:** 10.3390/pathogens10010065

**Published:** 2021-01-13

**Authors:** Katarzyna Morka, Ewa Wałecka-Zacharska, Justyna Schubert, Bartłomiej Dudek, Anna Woźniak-Biel, Maciej Kuczkowski, Alina Wieliczko, Jarosław Bystroń, Jacek Bania, Gabriela Bugla-Płoskońska

**Affiliations:** 1 Department of Food Hygiene and Consumer Health Protection, Wrocław University of Environmental and Life Sciences, C. K. Norwida 31, 50-375 Wrocław, Poland; ewa.walecka@upwr.edu.pl (E.W.-Z.); justyna.schubert@upwr.edu.pl (J.S.); jaroslaw.bystron@upwr.edu.pl (J.B.); jacek.bania@upwr.edu.pl (J.B.); 2 Department of Microbiology, Institute of Genetics and Microbiology, Wroclaw University, S. Przybyszewskiego 63, 51-148 Wrocław, Poland; bartlomiej.dudek@uwr.edu.pl; 3 Department of Epizootiology and Clinic of Birds and Exotic Animals, Wrocław University of Environmental and Life Sciences, pl. Grunwaldzki 45, 50-366 Wrocław, Poland; anna.wozniak-biel@upwr.edu.pl (A.W.-B.); maciej.kuczkowski@upwr.edu.pl (M.K.); alina.wieliczko@upwr.edu.pl (A.W.)

**Keywords:** virulence-associated genes, wild boars, *Yersinia enterocolitica*

## Abstract

*Yersinia enterocolitica,* widespread within domestic and wild-living animals, is a foodborne pathogen causing yersiniosis. The goal of this study was to assess a genetic similarity of *Y. enterocolitica* and *Y. enterocolitica*-like strains isolated from different hosts using Multiple Locus Variable-Number Tandem Repeat Analysis (MLVA) and Pulsed-Field Gel Electrophoresis (PFGE) methods, and analyze the prevalence of virulence genes using *multiplex*-Polymerase Chain Reaction (PCR) assays. Among 51 *Yersinia* sp. strains 20 virulotypes were determined. The most common virulence genes were *ymoA*, *ureC*, *inv*, *myfA*, and *yst*. *Yersinia* sp. strains had genes which may contribute to the bacterial invasion and colonization of the intestines as well as survival in serum. One wild boar *Y. enterocolitica* 1A strain possessed *ail* gene implying the possible pathogenicity of 1A biotype. Wild boar strains, represented mainly by 1A biotype, were not classified into the predominant Variable-Number Tandem Repeats (VNTR)/PFGE profile and virulotype. There was a clustering tendency among VNTR/PFGE profiles of pig origin, 4/O:3, and virulence profile. Pig and human strains formed the most related group, characterized by ~80% of genetic similarity what suggest the role of pigs as a potential source of infection for the pork consumers.

## 1. Introduction

The genus *Yersinia* has been recently classified to *Yersiniaceae* family [1] and comprises of 19 species, i.e., *Y. aldovae*, *Y. aleksiciae*, *Y. bercovieri*, *Y. enterocolitica*, *Y. entomophaga*, *Y. frederiksenii*, *Y. intermedia*, *Y. kristensenii*, *Y. massiliensis*, *Y. mollaretii*, *Y. nurmii*, *Y. pekkanenii*, *Y. pestis*, *Y. philomiragia*, *Y.pseudotuberculosis*, *Y. rohdei*, *Y. ruckeri*, *Y. similis*, and *Y.wautersii* with a wide range of hosts (fish, insects, plants, mammals, and humans) [1,2,3]. Species. i.e., *Y. pestis*, *Y. enterocolitica* and *Y. pseudotuberculosis* are pathogenic to humans. *Y. enterocolitica* and *Y. pseudotuberculosis* cause human yersiniosis commonly reported in Europe [4]. All *Y. pseudotuberculosis* strains are considered pathogenic, while *Y. enterocolitica* includes both pathogenic and nonpathogenic strains according to the biotypes division (1A, 1B, and 2–5). The most common bioserotypes causing human yersiniosis in Europe are *Y. enterocolitica* 4/O:3 and 2/O:9 [5,6]. Strains belonging to biotype 1A are considered to be nonpathogenic because they lack significant virulence genes such as the chromosomal *ail* gene (attachment-invasion-*locus*) and the 70-kb virulence plasmid pYV. However, there are reports about potential enteropathogenicity of strains from 1A biotype [7,8,9,10,11]. *Y. enterocolitica* is most commonly found in pig meat and its products, in particular in raw or undercooked pork and pork products, but also in milk and other dairy products, plants, seafood, and drinking water. Food, especially pork, since fattening pigs are a symptomless reservoir, may be contaminated primarily or cross-contaminated by infected surface or equipment used during food processing. Other sources of possible *Y. enterocolitica* contamination include environment, poultry, cattle, sheep, goats, cats, dogs, and wild animals such as rodents, deer, and boars. *Y. enterocolitica* is present in pigs’ tongues, oral cavities, tonsils, lymph nodes, and feces. During the slaughter and processing of meat, *Y. enterocolitica* may be transferred from contaminated tissues onto the rest of the pig carcass and next persists in cooling conditions in pork products in retailers [12,13,14]. The risk factors of *Y. enterocolitica* transmission are poor hygiene after batch removal and the absence of cloth-boot change rooms at entrances to the facilities, straw on floors or mixing pig batches [15]. Furthermore, *Y. enterocolitica* strains are not detectable within the current pig meat inspection in EU [16]. The remaining species of the *Yersinia* genus were noted in the environmental samples, i.e., *Y. pekkanenii* was previously isolated from plants, water, and soil samples which indicates the environmental occurrence [17]. *Y. kristensenii* and *Y. frederiksenii* also tested within this work (listed in Table 1) have been isolated primarily from fresh water, sewage, soil, fish, wild rodents, domestic animals, foods, healthy and sick humans [18].

Many known virulence determinants are present in pathogenic biotypes of *Y. enterocolitica*. The most important for *Yersinia* sp. virulence are YadA (*Yersinia* adhesin A, outer membrane protein) conferring bacteria human serum resistance and ability to adhere to the intestinal epithelium; Ail (outer membrane protein) ensuring adhesive-invasive properties and invasin Inv (transmembrane protein) enabling rapid transcytosis through the intestinal epithelial layer. A thermostable enterotoxin Yst causes damage of the intestinal mucosa resulting in diarrhea in humans. An important chromosomal gene is *ymoA* (*Yersinia*-modulating protein) encoding the YmoA protein, which negatively regulates the expression of various genes, e.g., *inv* and *ystA*. Another virulence factor, a Myf antigen [8,19] is present in pathogenic biotypes and it possibly participates in the colonization of the gut, production of enterotoxin Yst, and protection against phagocytosis. The type III Yop effector proteins secretion system (T3SS) enables pathogenic *Yersinia* species to survive in the extracellular environment such as human intestine. A less-known type II secretion system (T2SS) probably plays a dual role for both the pathogenicity and the environmental survival of *Y. enterocolitica*. [20,21]. Due to this, in this work, the presence of the T2SS (Yts1) was screened in all tested *Yersinia* sp. More details on the other *Yersinia* sp. virulence factors can be found in [22,23,24,25].

Virulence genes such as *myfA*, *invA* and *ymoA* (Supplementary Table S1), characteristic of the pathogenic strains of *Y. enterocolitica* [8,26], have been detected in the genome of strains of 1A biotype [8]. This shows that the division of biotypes may not pertain the real pathogenicity within the *Y. enterocolitica* species. Due to this fact, the assessment of virulence profiles of *Y. enterocolitica* and *Y. enterocolitica*-like strains, i.e., *Y. kristensenii* or *Y. frederiksenii* and *Y. pekkanenii* is required. 

The *Y. enterocolitica* occurrence in wild boars in Poland has so far been confirmed by Morka K. et al. in western Poland in 2018 [27], Syczyło et al. in 2016 [28] during 2011–2014 hunting seasons mainly in North-Eastern Poland, Syczyło et al. in 2018 [29] in 12 out of 16 Polish districts across Poland during the hunting seasons of 2013–2014, Bancerz-Kisiel et al. in 2015 [30] in North-East Poland during the 2013 hunting season and Bancerz-Kisiel et al. in 2015 [31] in two voivodeships of northern Poland, one voivodeship of central Poland and from two voivodeships of southern Poland. Only one paper, a published dissertation, indicated the endemic or alluvial origin of *Y. enterocolitica* strains isolated from people in the years 1996–2008 [32]. Nevertheless, data on the relatedness of *Y. enterocolitica* strains present among the animal reservoir or their similarities to human strains are still missing in Poland. The genetic comparison of *Yersinia* sp. strains isolated from pigs, wild animals, and humans will reveal the phylogenetic relationships between strains from varied origins. 

The main object of this work was to determine the virulotypes and genetic profiles of *Y. enterocolitica* and *Y. enterocolitica*-like strains isolated from both humans and animals. To determine the genetic relationships between strains the gold standard PFGE (Pulsed-Field Gel Electrophoresis) and MLVA (Multiple Locus Variable-Number Tandem Repeat Analysis) were used to generate a DNA fingerprint of a single bacterial isolate.

## 2. Results

### 2.1. Virulotyping

**Figure 1:**
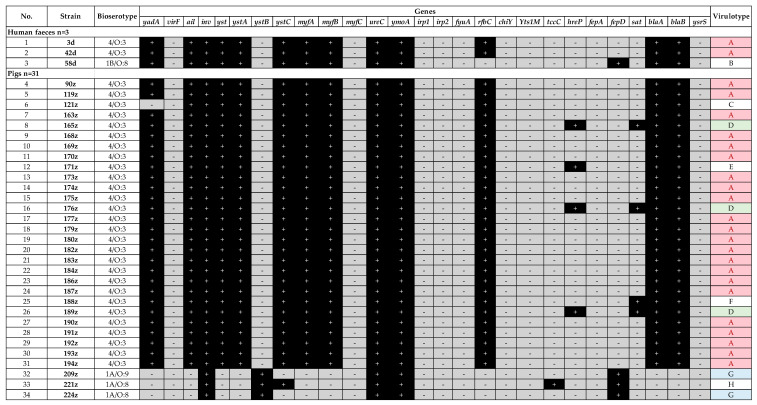
Virulence profiles of *Y. enterocolitica* strains isolated from human feces and pigs.

**Figure 2:**
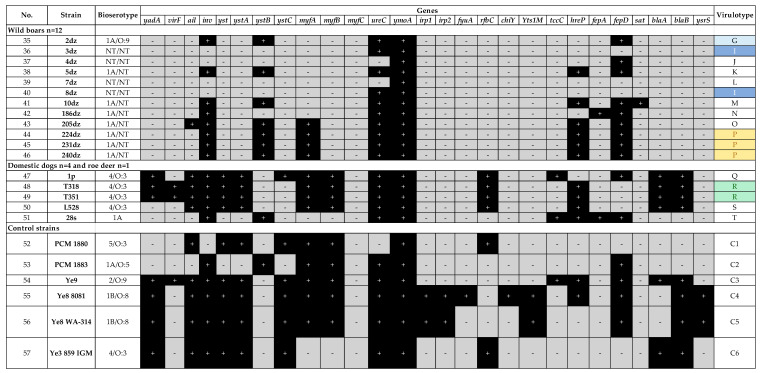
Virulence profiles of *Yersinia* sp. strains isolated from wild boars, dogs, roe deer, and control strains.

The presence of frequent *Yersinia* virulence genes was assessed in this study. In this work 20 different virulence profiles (designated from A to T) were determined among 51 *Yersinia* sp. strains (Figure 1 and Figure 2). The most common virulence gene present in all strains was *ymoA*, a *Y. enterocolitica* chromosomal gene modulating the expression of virulence functions (n = 51/51), followed by *ureC* coding the enzyme urease (n = 49/51), *inv* coding the invasive protein (n = 47/51), *myfA* coding Myf antigen involved in the colonization of the intestine (n = 39/51) and *yst* coding the enterotoxin (n = 35/51). 

The same profile A was observed for two out three human *Y. enterocolitica* strains (3d and 42d, 4/O:3, Figure 1). Another human strain (58d, 1B/O:8) lacked 4/O:3-specific *rfbC* gene, but possessed *fepD* gene coding enterochelin ABC transporter – profile B (Figure 1). This strain lacked the *irp1*, *irp2*, *chiY*, and *Yts1M* genes found in the reference strain of the same bioserotype 1B/O:8 (Figure 2).

Profile A was the most numerous within the pig *Y. enterocolitica* strains (n = 22/31, 71%) as among the human isolates. Virulotypes G and D were represented by 6.5% (n = 2/31) and 9.7% (n = 3/31) of pig strains, respectively; while each of the C, E, F, and H profiles was associated with only one pig strain (n = 1/31, 3.2%). The differences between profiles among pig strains were related to *hreP* (subtilisin/kexin-like protease), *sat* (streptogramin acetyltransferase), *fepD* (enterochelin ABC transporter) or *ystB* (enterotoxin YstIB of nonpathogenic biotype 1A) genes (Figure 1).

Nine virulotypes were noted within the wild boar *Yersinia* sp. strains, i.e., P (n = 3/12); I (n = 2/12) and L, J, G, K, M, O, N represented only by a single strain (n = 1/12). A very important observation is the presence of *ail* – adhesion-invasion and serum resistance-associated gene in *Y. enterocolitica* 205dz strain (1A biotype) (Figure 2). 

The remaining *Y. enterocolitica* strains, which were isolated from dogs (4/O:3) and roe deer, were of different virulotypes, when compared to the other strains. Profiles Q and S were represented by single dog strains (n = 1/4), while profile R by two strains (n = 2/4). Roe deer 28s strain had profile T (Figure 2).

The less frequent genes among all tested strains were *tccC*, *hreP*, *fepA*, *fepD*, and *sat* (Figure 1 and Figure 2). Gene *tccC* coding an insecticidal toxin complex protein was present only in several isolates, i.e., 1A/O:8 pig (221z, n = 1/31), 4/O:3 dog (1p, n = 1/4) and in wild-living roe deer (28s) isolates. Gene *hreP* (subtilisin/kexin-like protease) was present within pig (n = 4/31), dog (n = 3/4), wild boar (n = 6/12) and one roe deer (28s) isolates. Gene *fepD* (enterochelin ABC transporter) was detected within one human 1B/O:8 isolate (n = 1/3), pig 4/O:3 isolates (n = 3/31), wild boar isolates (n = 9/12) and one roe deer isolate, while gene *fepA* was only present in wild boar 186dz isolate (n = 1/12) and roe deer isolate (28s). Gene *sat* (streptogramin acetyltransferase) was identified only in n = 4/31 pig 4/O:3 isolates and one n = 1/12 wild boar isolate.

### 2.2. Tracking Potential Variable-Number Tandem Repeats—VNTRs and Cluster Analysis

To characterize the group of n = 51 *Yersinia* sp. strains according to their genetic relationship the Tandem Repeats Finder was used to detect sequences containing VNTRs (variable-number tandem repeats) within the *Y. enterocolitica* 8081 genome. These VNTRs, met the criteria for easy handling, i.e., a repeat length greater than 20 nucleotides and at least 2 copies with a highly conserved repeated sequence (>90% matches). In this work only 5 of the 28 detected VNTRs *loci* (A-E) were selected for further analysis (Table 2). The number of tandem repeats was calculated based on previously described scheme in methodology and then used in clustering and listed in a phylogenetic tree.

**Figure 3:**
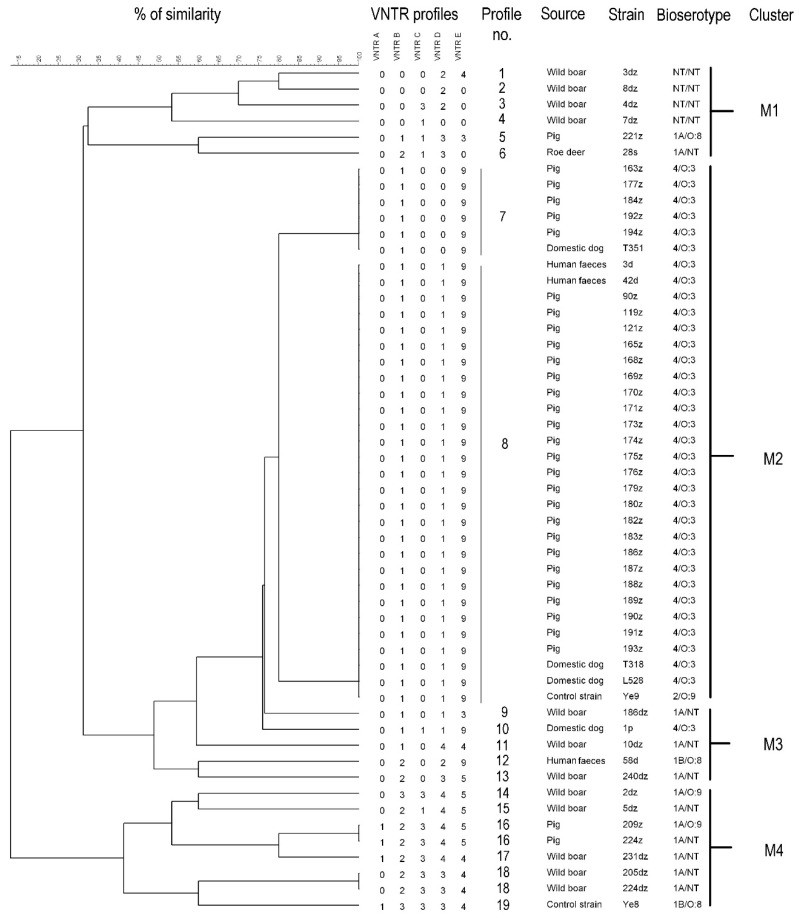
Cluster analysis of MLVA profiles of *Yersinia* sp. with their corresponding origin and bioserotype. The tree was generated by the average linkage agglomeration method (unweighted pair group method with arithmetic mean) based on the calculated number of tandem repeats using BioNumerics 7.6.2 (Applied Maths).

Within the 53 tested *Yersinia* sp. strains (including two control strains) the total number of VNTR profiles was 19. According to the phylogenetic tree these profiles formed four main clusters. Cluster similarity cut-off value was set at least at 30% (Figure 3). The Simpson’s index of diversity for MLVA clustering was 0.6074. The same profile had twenty-eight 4/O:3 strains and one 2/O:9 control strain. Within this profile n = 23/28 strains were isolated from fattening pigs; n = 2/28 from domestic dogs; n = 2/28 from human feces and n = 1/28 was a control strain. The following profile represented by 4/O:3 bioserotype counted n = 5 strains isolated from fattening pigs and n = 1 from domestic dog. These two profiles shared 80% of genetic similarity and formed the M2 cluster (Figure 3). The M1 cluster consisted of wild animals (wild boar and roe deer) and pig strains being characterized by ~35% similarity, whereas M3 consisted of wild boar, human feces and dog strains sharing ~49% similarity. The last M4 cluster with ~41% similarity contained wild boar, pig, and control strains. (Figure 3). 

### 2.3. PFGE-CHEF (Contour-Clamped Homogeneous Electric Field)

**Figure 4:**
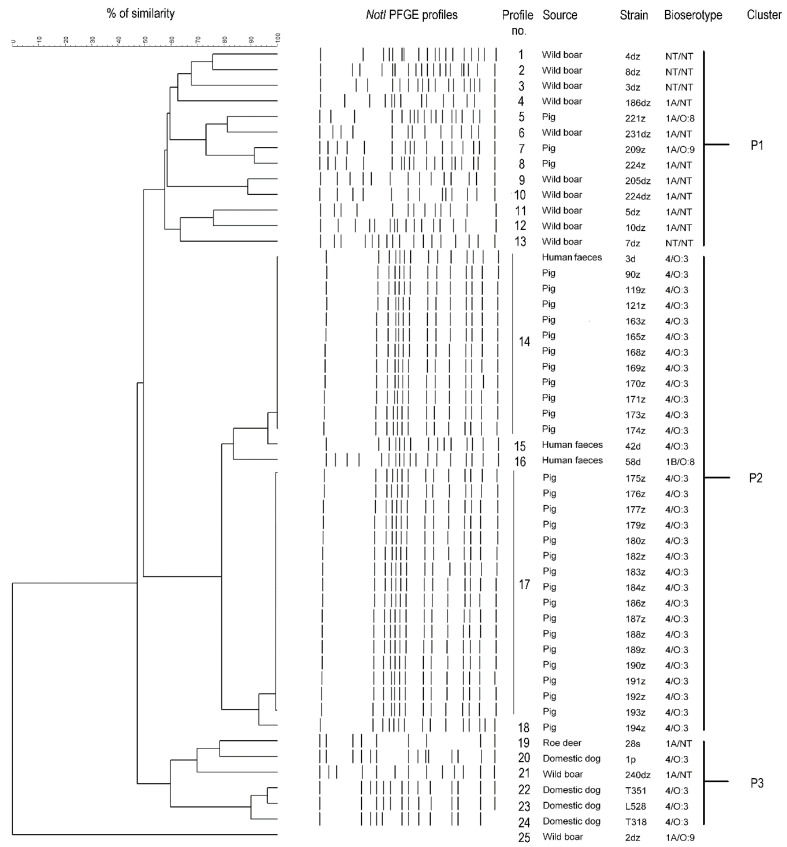
Cluster analysis of PFGE profiles of *Yersinia* spp. with their corresponding origin and bioserotype. The tree was generated by the average linkage (unweighted pair group method with arithmetic mean) based on the PFGE profiles using BioNumerics 7.6.2 (Applied Maths).

Within the 51 tested *Yersinia* sp. strains the total number of PFGE profiles was 25. According to the phylogenetic tree these profiles formed three main clusters. Cluster similarity cut-off value was set at least at 60% The overall Simpson’s index of diversity for PFGE-CHEF clustering was 0.706 (Figure 4). 

The P2 cluster with 80% genetic similarity included five PFGE profiles, i.e., n = 12 4/O:3 strains (isolated from pigs and human feces), n = 16 4/O:3 strains (isolated from pigs) and *Y. enterocolitica* 42d, 58d and 194z strains (Figure 4). Other profiles (n = 20/25) represented by a single wild animal, pig, roe deer and dog strains formed the P1 and P3 clusters with ~60% genetic similarity. The 2dz strain was not assigned to any of mentioned clusters however, it is shown on the phylogenetic tree as an unbound branch (Figure 4).

## 3. Discussion

The causative agent of yersiniosis is *Yersinia enterocolitica*, an important food- and water-borne enteropathogen, belonging to the *Yersiniaceae* family. Between 2014–2018 the number of yersiniosis cases in Poland has remained at constant levels (incidence rate between 0.4–0.6) [33]. In the years 2003–2019, the highest number of yersiniosis cases (n = 326) was recorded in 2009 [34]. Nevertheless, data on the number of cases can be underestimated due to low reportability and lack of bioserotyping as a part of routine diagnostics. In Poland prevalence of *Y. enterocolitica* and other species within *Yersinia* genus among animals was only studied in Northeast Poland [26,28,29,31,35]. High prevalence of *Y. enterocolitica* in the world, high pathogenicity of the 1B biotype, unusual psychrotrophic features and not much information on the occurrence of *Yersinia* sp. throughout the territory of Poland justify the study of *Y. enterocolitica* occurrence and its virulence genes. Except for *Y. enterocolitica*, *Y. pseudotuberculosis* and *Y. pestis*, there are also other species, ignored so far, which may be potentially pathogenic to humans or animals [18]. In the current work three *Y. enterocolitica*-like strains were included, i.e., *Y. kristensenii*, *Y. frederiksenii* and *Y. pekkanenii*. 

The main source of *Y. enterocolitica* infection in humans is still pork and pork products, while *Y. frederiksenii*, *Y. kristensenii* and *Y. pekkanenii* have been isolated primarily from various environmental samples (water, sewage, soil), foods (milk, fruit, vegetables), domestic, and wild animals as well as healthy, and sick humans [17,18]. *Y. enterocolitica* is transmitted from slaughterhouses to meat-processing plants and then to retails via contaminated pig carcasses. It is facilitated by bacterial ability to multiply at low temperatures, even in frozen foods, and in a pH ranging from 4.2 to 9.0. Virulence factors such as pYV plasmid and YadA as well as *ureC* confer the resistance to low pH [36]. Due to the psychrotrophic feature and ability to produce the enterotoxin both at 37 °C (at pH 7.5) and at temperatures below 30 °C, *Y. enterocolitica* can survive cooling conditions and cause symptoms of diarrhea in humans after eating contaminated food [37]. 

A new tested reservoir for *Y. enterocolitica* includes wild animals such as wild boars or roe deer, which is reasonable considering that venison consumption is becoming increasingly popular [38]. The common assumption that only *Y. enterocolitica*, *Y. pseudotuberculosis* and *Y. pestis* are clinically relevant and the remaining species are environmental may have an impact on the identification of potential new hosts that can cause human disease. This also leads to overrepresentation of clinical *Y. enterocolitica* strains in databases and a limited number of representative *Y. enterocolitica*-like species in matrix-assisted laser desorption/ionization (MALD)I Biotyper or VITEK^®^ 2 Compact (bioMérieux) databases, which together require an application of several identification methods simultaneously, what was highlighted in a recently published paper [27]. 

In this study, virulence profiles and genetic relationships of strains derived from pig, wild boar, dog, roe deer, and human feces samples were investigated. The strains were screened for 27 virulence-associated genes, i.e., *yadA*, *virF*, *ail*, *yst*, *ystA*, *ystB*, *ystC*, *ysrS*, *myfA*, *myfB*, *myfC*, *irp1*, *irp2*, *fyuA*, *Yts1M*, *chiY*, *inv*, *tccC*, *hreP*, *fepA*, *fepD*, *sat*, *blaA*, *blaB*, *rfbC*, *ureC*, and *ymoA.* It was found that *Y. enterocolitica* strains isolated from pigs were characterized by less diversity of virulotypes and VNTR/PFGE profiles in contrast to wild boar group of strains. (Figure 1 and Figure 4). Most of the 4/O:3 strains isolated from fattening pigs slaughtered in west Poland carried virulence determinants and displayed the same virulence genes pattern between them, suggesting that pigs are an important reservoir of *Y. enterocolitica* 4/O:3 isolates (Figure 1). This observation is convergent with previous literature reports [39,40,41]. An exception for this remark was *Y. enterocolitica* 121z strain lacking *yadA* gene, which can be explained by the loss of pYV plasmid due to standard conditions for bacterial cultivation (incubation at 37 °C) [42,43]. Interestingly, in this paper there are some strains possessing additional genes such as *sat* (streptogramin acetyltransferase) and *hreP* (subtilisin/kexin-like protease) (Figure 1) previously reported as important virulence factors in *Y. enterocolitica* 1B and 1A biotypes, *Y. pestis* and *Y. ruckeri* [9]. The lack of *virF* gene in the majority of tested strains has been reported before [41,44]. 

This work described n = 12/51 1A *Y. enterocolitica* strains isolated from fattening pigs, wild boars and roe deer (Table 1) widely considered to be nonpathogenic to humans. However, in the last century, the first reports on nosocomial infections of *Y. enterocolitica* 1A were published [45,46]. Among the tested 1A group of strains, 11 had the *ystB* gene (Figure 1 and Figure 2). This gene is considered to be commonly present among nonpathogenic strains and its function is to control the production process of the heat stable enterotoxin. Although *ystB* gene does not encode an enterotoxin causing a damage to the intestinal epithelium there are reports demonstrating that *Y. enterocolitica* biotype 1A with the *ystB* gene is more often isolated from stool samples of patients suffering from diarrhea [37]. This information indicates that some strains of *Y. enterocolitica* biotype 1A are able to cause gastrointestinal symptoms after ingestion of contaminated food [8,9,10,11,47,48,49,50]. Altogether these findings indicate the role of the YstB toxin in the pathogenicity of *Y. enterocolitica* and suggest that biotype 1A cannot be considered completely nonpathogenic to humans. Despite the *ystB* gene, other genes had been detected within the tested *Y. enterocolitica* 1A isolates, i.e., *ureC* (12/12), *fepD* (12/12), *tccC* (2/12), *sat* (1/12), and *hreP* (7/12) (Figure 1 and Figure 2). The genes *ureC* and *tccC* enable bacteria to survive in the stomach and gastrointestinal tract, *sat* confers resistance to streptogramins, which inhibit protein synthesis by ribosomes, while *hreP* is associated with early stage of 1B/O:8 infection [9,10]. 

It is noteworthy that *Yersinia* sp. strains (n = 12) isolated from wild boars harbor virulence-associated genes such as: *inv*, *ystB*, *ureC*, *ymoA*, *hreP* and *fepD* (Figure 2) and belong to 1A biotype. Moreover, one strain named 205dz (Table 1, 1A/NT) possessed *ail* gene, conferring bacteria human serum resistance and adhesive-invasive properties, characteristic of strains from pathogenic biotypes, what strongly supports an assumption about potential pathogenicity of 1A strains (Figure 2). Other papers also indicated the *ail* gene presence in 1A *Y. enterocolitica* strains [11,44,48,51,52,53]. One of them, recently published in Poland by Platt-Samoraj et al. [11] in 2017 proved that *Y. enterocolitica* 1A *ail*-positive strains are widely distributed in the environment. Batzilla et al. in 2011 confirmed that *Y. enterocolitica* 1A could be an emerging and opportunistic pathogen whose ability to cause an infection state seems to be dependent mainly on the host defense system [8]. These papers showed potential pathogenicity and ability of 1A strains to cause disease in humans. The presence of *ail* gene within nonpathogenic strains of 1A biotype could be recognized as the beginning of their acquisition of virulence-associated genes questioning biotypes division in terms of pathogenicity. On the other hand, Sihvonen et al. in 2011 compared the sequence of the *ail* gene from biotype 1A with *ail* derived from 1B indicating one point mutation—transition G2008088T and 99.7% of similarity, which according to the authors is the basis for studying the functionality of the *ail* gene in 1A strains. [48]. This suggests that molecular analyses of the pathogenicity should not be performed only based on possessing the virulence-associated *ail* gene. Taken together, the use of one method in determining potential pathogenicity may be insufficient, therefore, both virulotyping and bioserotyping seem to be worth an effort. 

Currently, only limited data is available on the possible virulence, relatedness, and reservoir role of animals in human yersiniosis. Thus, the goal of this study was to analyze the genetic diversity of *Yersinia* sp. strains isolated from its reservoir—wild-living animals and fattening pigs and evaluate their genetic relationship with human *Yersinia* sp. strains. To reach this objective the *multiplex*-PCR, PFGE-CHEF, and MLVA were used. PFGE-CHEF is still a gold standard in epidemiological investigations while MLVA allows detection of more detailed differences. The advantage of PFGE is its adjustment to the specific tested microorganism. Despite this, Gilpin et al. claimed that PFGE has a very limited application for the discrimination of *Y. enterocolitica* biotypes 2, 3, and 4, but it is still valid for biotype 1A isolates [54]. It should be taken into account that current methods such as cgMLST or wgMLST, which, despite the high costs are less labor-intensive and more discriminatory techniques than PFGE or MLVA [54]. 

The presented study showed the clustering tendency among PFGE and VNTR profiles related to pig origin, 4/O:3 bioserotype and virulence profile and the similarity of PFGE and VNTR profiles between human and pig strains. Genetic and virulotype similarity between human and pig strains determined in this work strongly correlate with recognition fattening pigs and their meats as a main risk factor for transmission of *Y. enterocolitica* [55]. The same was shown in the studies conducted in Finland and Germany, which proved the genetic relationship of 81% and 83% of tested clinical strains with pig isolates in Finland and Germany, respectively [56,57]. Similar results were obtained by comparing the genetic relationship of strains isolated from humans, pigs, purchased pork, offal and domestic animals in Finland [56]. In contrast, there was no similarity between pig strains isolated in Finland and Germany, which was considered the effect of geographic distribution [57]. So far only Fredriksson-Ahomaa et al. proved significant differences between isolates from wild boars and slaughter pigs, which was also observed in our results—wild boar only represented biotype 1A while pig’s isolates mainly biotype 4/O:3 [58]. 

This work characterized *Y. enterocolitica* and *Y. enterocolitica*-like strains in terms of genetic similarity and virulence genes content. Presented results are certainly the basis for continuing research on the pathogenicity of *Yersinia* sp.

## 4. Materials and Methods 

### 4.1. Bacterial Strains

A total of 51 tested *Yersinia* sp. strains originated from human stool (n = 3, Lower Silesia voivodship), wild boars (n = 12, Greater Poland voivodship), domestic dogs (n = 4, Lower Silesia voivodship), roe deer (n = 1, Lower Silesia voivodship) and fattening pigs (n = 31, Lower Silesia and Greater Poland voivodships), isolated in Poland during the years 2014–2018 were included in this study (Table 1). All tested strains were identified using MALDI TOF MS, VITEK^®^2 Compact (bioMérieux), PCR targeting 16S rRNA of *Y. enterocolitica*, 16S rRNA sequencing, and bioserotyped as previously described [27]. 

The *Y. enterocolitica* reference strains used as controls in this study were obtained from Polish Collection of Microorganisms (PCM, Wroclaw, Poland): PCM 1879 (4/O:3), PCM 1880 (5/O:3), PCM 1881 (4/O:3), PCM 1882 (1B), PCM 1883 (1A/O:5), PCM 1884 (2/O:8) and from Department of Applied Microbiology, Faculty of Biology, University of Warsaw, Poland: *Y. enterocolitica* 2/O:9, *Y. enterocolitica* WA-314, *Y. enterocolitica* 8081.

### 4.2. DNA Isolation

Bacteria were grown in Luria-Bertani Broth (Biocorp, Warszawa, Poland) for 16 h at 28 °C. Then 1 mL of bacterial suspension was centrifuged at 10,000 rpm, for 10 min at room temperature. Extraction of genomic DNA was carried out using mini-columns from Genomic Mini kit (A&A Biotechnology, Gdańsk, Poland), according to the manufacturer’s protocol. 

### 4.3. Detection of Virulence-Associated Genes of Yersinia sp.

Several *multiplex*-PCRs based on gene grouping were used to detect virulence-associated determinants as: *ail*, *ystB*, and *ymoA*; *blaA* and *blaB*; *chiY*, *ysrS*, and *yst1M*; *fepA* and *fepD*; *myfA*, *myfB* and *myfC*; *ureC* and *irp1*; *sat* and *hreP*; *rfbC* and *tccC*; *virF*, *invA* and *yst*; *irp2*, *ystA*, and *16S*; *yadA* and *ystC*; *fyuA* (Table S1). The 16S rDNA gene (amplified by following primers: AATACCGCATAACGTCTTCG and CTTCTTCTGCGAGTAACGTC) was used as a control specific for *Y. enterocolitica* [59]. 

The PCR mixtures consisted of 1 mM MgCl_2_, 1 × buffer, 0.2 mM dNTP, primers (Genomed, Warszawa, Poland) with a concentration in the range of 0.15 to 1.25 µM as indicated in Table S1, 1U of *Taq* polymerase, and 1 µL of template DNA. PCRs were performed with an initial denaturation step at 94 °C for 3 min, 35 cycles each of denaturation, annealing and extension as presented in Table S1 and a final extension of 10 min at 72 °C. Amplicons were separated in 1.5% agarose gel containing 0.5 μg/mL ethidium bromide, at 120–130 V for 1 h and documented using GelDocXR System (Bio-Rad, Hercules, CA, USA) [35,60].

### 4.4. Multiple Locus Variable-Number Tandem Repeat (VNTR) Analysis—MLVA

The genome of the *Y. enterocolitica* subsp. *enterocolitica* 8081NC_008800 was scanned for tandem repeats using the Tandem Repeats Finder program (version 2.02 by Gary Benson) [61]. Five *loci* A, B, C, D, E were chosen for MLVA analysis. They were amplified by primers designed in this study (Table 2). PCRs were performed in a total volume of 25 µL containing 1.5 µL of 25 mM MgCl_2_, 2.5 µL of 10 × buffer, 0.5 µL of 10 mM dNTP, 0.1 µL of 100 µM primers (Genomed, Warszawa, Poland), 0.2 µL of *Taq* polymerase and 0.5 µL of DNA template. Thermocycling conditions included initial denaturation at 94 °C for 2 min and 35 cycles of: denaturation at 94 °C for 30 s, annealing at 55 °C for 60 s, and elongation at 72 °C for 120 s followed by final extension at 72 °C for 3 min. Amplification products were resolved in 2.5% agarose containing 0.5 μg/mL ethidium bromide in Tris-Acetate Electrophoresis 1× (TAE) buffer (pH 8.0), at 180 V for 2 h with cooling and documented using GelDoc XR System (Bio-Rad, Hercules, CA, USA).

Each product after electrophoresis was sequenced (Genomed, Warszawa, Poland). The number of repeats was calculated by dividing the difference of amplicon length and offsets size by the size of repeat, determined by BioNumerics 7.6.2 (Applied Maths). Dendrogram was generated using BioNumerics 7.6.2 (Applied Maths) by unweighted pair group method with arithmetic mean (UPGMA) clustering and categorical coefficient with a tolerance of 0%. The discriminatory power was calculated with the Simpson’s index of diversity [62].

### 4.5. Pulsed-Field Gel Electrophoresis—Contour-Clamped Homogenous Electric Field—PFGE-CHEF

The *Yersinia* sp. DNA plugs were prepared in Cell Suspension Buffer (CSB) according to PulseNet *Yersinia pestis* protocol with small modifications (www.pulsenetinternational.org/protocols) [63]. Briefly, the plugs were lysed for 2 h at 51 ± 2 °C in CLB/proteinase K (0.1 mg/mL) solution. The plugs were washed twice in pre-heated (50 °C) sterile water and four times in Tris-EDTA (TE) buffer (pH 8.0) before restriction digestion. Bacterial DNA trapped in low melt 1.0% agarose (ABO, Gdańsk, Poland) was digested with 5U of *NotI* at 37 °C for 2 h. The restriction fragments were separated using 1.0% gel in 0.5 × TBE buffer with a CHEF Mapper XA system (Bio-Rad, Hercules, CA, USA). The electrophoresis was carried out under following conditions: initial switch time: 3 s, final switch time: 30 s, voltage: 6 V/cm, included angle: 120°, run time: 20 h, temp. 14 °C. The gels were stained with ethidium bromide, destained with ultrapure water, and photographed with a Gel Doc EQ system (Bio-Rad, Hercules, CA, USA). The DNA bands corresponded to specific PFGE profiles for each strain. Dendrogram was generated using BioNumerics 7.6.2 (Applied Maths) by Dice coefficient with optimization of 0.5% and an UPGMA clustering with a tolerance of 1.0%. The discriminatory power was calculated with the Simpson’s index of diversity [62].

## 5. Conclusions

The results of this study confirm that *Y. enterocolitica* strains isolated from pigs were less diverse in terms of virulotypes and VNTR/PFGE profiles in contrast to wild boar- derived group of strains. Wild boar strains represented only by biotype 1A and pig isolates mainly by biotype 4/O:3 were significantly different according to their genetic profiles. Pigs are an important host of *Y. enterocolitica* 4/O:3- carrying virulence determinants and this result has valuable implications in terms of food safety. Since fattening pigs are asymptomatic reservoirs (no clinical symptoms nor gross pathological lesions are visible) *Y. enterocolitica* strains are not detectable within the current pig meat inspection in EU. Additionally, *Y. enterocolitica* strains of biotype 1A can pose a risk of yersiniosis since they cannot be considered completely not virulent.

## Figures and Tables

**Table 1:** *Yersinia* spp. strains (n = 51) used in this study.

Species	Origin (Number of Isolates)	Bioserotype—Strain Name
*Y. enterocolitica*	Human feces (n = 3)	4/O:3—3d, 42d 1B/O:8—58d
	Fattening pig feces (n = 28)	4/O:3—163z, 165z, 168z, 169z, 170z, 171z, 173z, 174z, 175z, 176z, 177z, 179z, 180z, 182z, 183z, 184z, 186z, 187z, 188z, 189z, 190z, 191z, 192z, 193z, 194z 1A/O:9—209z 1A/O:8—221z, 224z
	Fattening pig tonsil (n = 3)	4/O:3—90z, 119z, 121z
	Wild boar tonsil (n = 8)	1A/NT—2dz, 5dz, 10dz, 186dz, 205dz, 224dz, 231dz, 240dz
	Domestic dog feces * (n = 4)	4/O:3—1p, T318, T351, L528
	Roe deer tonsil (n = 1)	1A/NT—28s
*Y. kristensenii*	Wild boar tonsil (n = 4)	3dz, 4dz
*Y. frederiksenii*		7dz
*Y. pekkanenii*		8dz

* Additional strains delivered by veterinary laboratory isolated from domestic dogs (Lower Silesia voivodship). *Y. kristensenii*, *Y. frederiksenii* and *Y. pekkanenii* were not bioserotyped by method for *Y. enterocolitica*. NT- not typeable.

**Table 2:** VNTR sequences and primers used in this study.

VNTR	Repeat Sequence	Primer Sequence (5′→3′)	Size of Repeat [bp]	Size of Offsets [bp]
A	GAGCCGCAGCGGTGTTAGCGGCTCTCACTTACCCGAATCACTTACCGTTGTAAGCTCATCGGGATGCATTCGCATGCTGCCTTGCTGCAACACCAATTACTTTGAGTAACATCTTTTTTAATAA	CATTGTATTACCCAGCAGATCC GGTGATACAGAGCGGTAGAC	124	177
B	CAGTAAGTGATTCGGGTAAGTGAGTGCAGTCAACACCGCTGCAACTTGAAAGATGACGGCATACCTCTATCCAATAGATTTCATGTTGCAGCAAGGCGGCAAACGAGATTATCCCGATGAGCTTACAT	ATTGACGGTCGGTATTATCTCG ACTAAGGTTTGGGGTGACATAC	128	167
C	TATTGGCTACATAAATAGATTAGCTACATAAATA	ATTATAGTGGCGGTCATTATCG AATAGCTTTCATCAAGCCTGTC	34	203
D	ATCTTCCTGGGTGCACCATCTTAAGAAACCTCGCTACGGCGGGGTTTTTCGTTTTTAGATTCTACAAATACGCAGAACCGAGCGGTCGGAAGTTCGAGCCGAGCGTAGCGAGACAACGTTGCTTTAGCAACGGCCCGCAGGGCGAGGCGTTAGCCGAGTC	GTCAAATGAACATCAGGTAGTG TCATTCACAATAGGAAATAGCG	160	204
E	TTGCGCGGCCTTTTTAGCTTTCACGCGAGCAATCGCTGCGGCCACGGCAGCTTTGCGCGGATCTTCCTCTGGCGCTTCACTTTCGCTTTTTACCGGTTCTGACTCAAGTTGAGC	GGAAGTGATTGAGGTTGATAGC GTTATTGCTGCTCGTGAGG	114	260

## Data Availability

The data presented in this study are available in Supplementary Materials.

## Supplementary Materials

The following are available online at https://www.mdpi.com/2076-0817/10/1/65/s1. References [64,65,66,67] are cited in the supplementary materials.
