# Supplementary material for: Genetic Diversity and Distribution of Virulence-Associated Genes in Y. enterocolitica and Y. enterocolitica-Like Isolates from Humans and Animals in Poland
Source: Pathogens. 2021 Jan 13;10(1):65. doi: 10.3390/pathogens10010065 (PMC7828411; doi:10.3390/pathogens10010065)
Supplement: Supplementary file 1 [file pathogens-10-00065-s001.pdf]

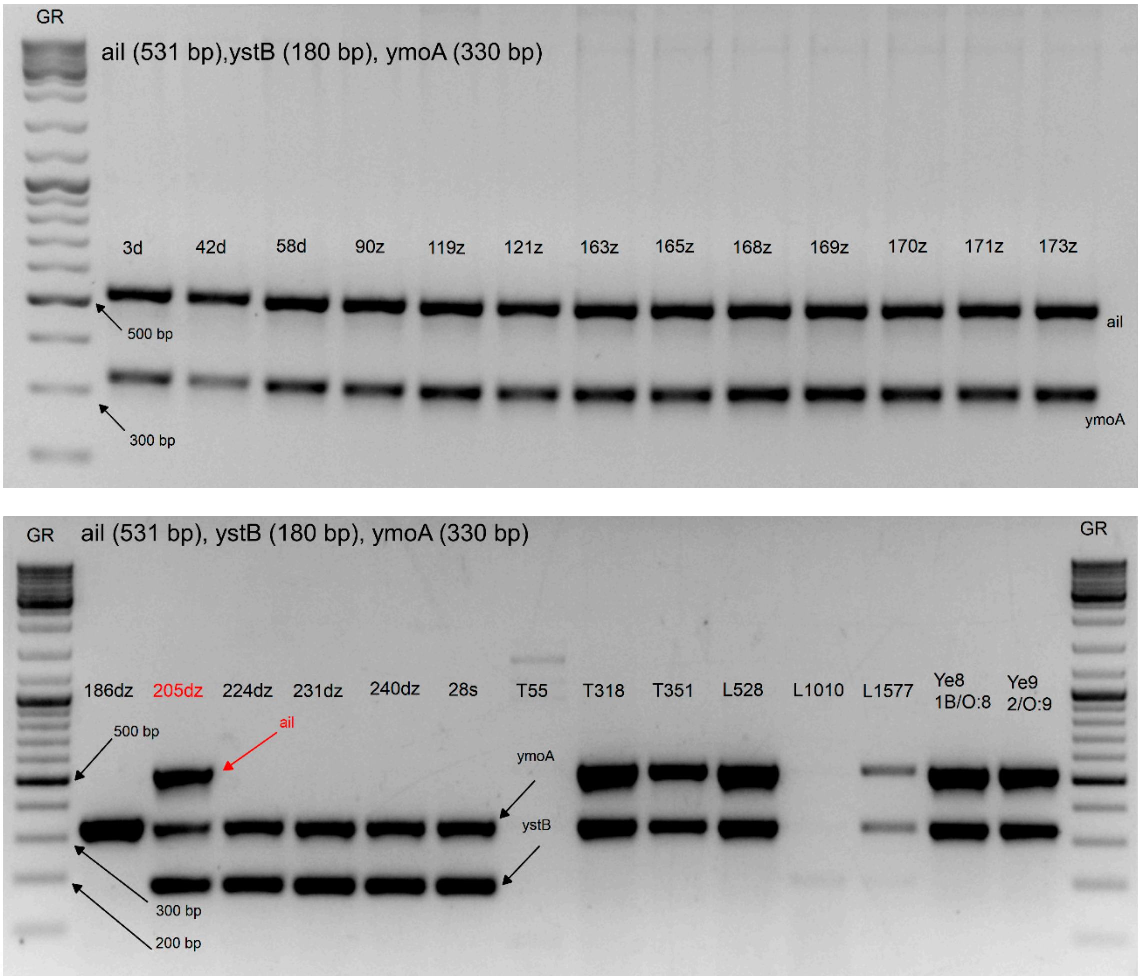

Figure S1. Gel electrophoresis after amplification of the *ail*, *ystB* and *ymoA* virulence genes of selected *Yersinia* sp. strains isolated from human faeces (3d-58d), fattening pigs (90z-173z), wild boars (186dz-240dz), roe deer (28s), dogs (T318, T351, L528) and reference strains (Ye8 1B/O:8, Ye9 2/O:9). Isolates T55, L1010, L1577 are not included in publication. GR- Gene RulerTM – DNA ladder.

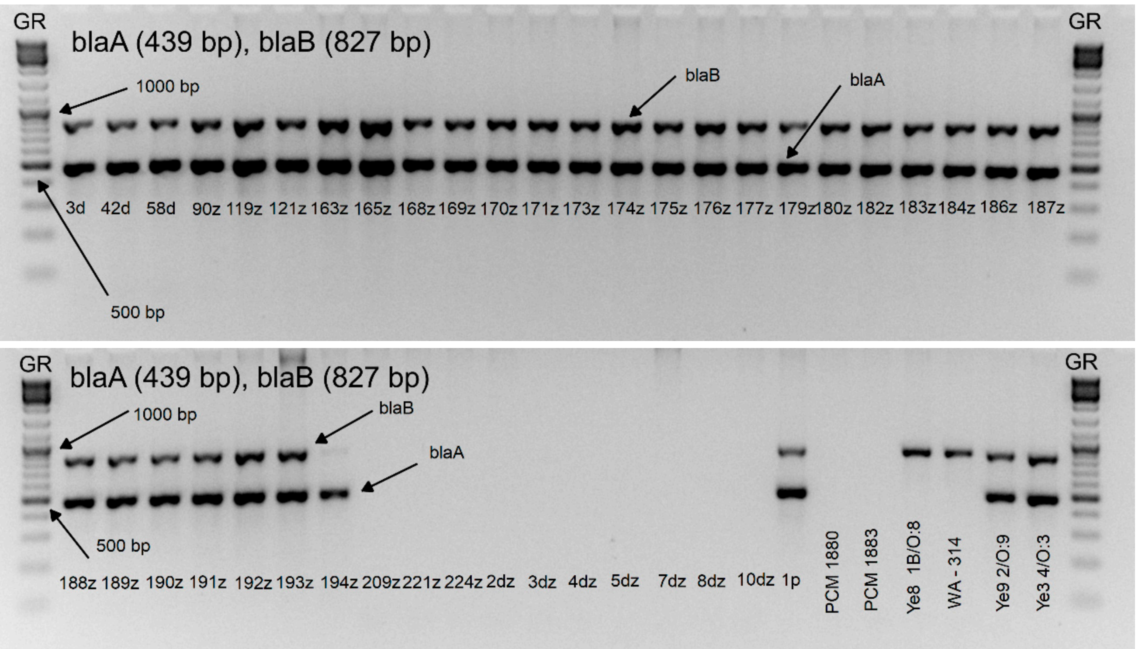

Figure S2. Gel electrophoresis after amplification of the *blaA* and *blaB* virulence genes of selected *Yersinia* sp. strains isolated from human faeces (3d-58d), fattening pigs (90z-224z), wild boars (2dz-10dz), dogs (1p) and reference strains (PCM 1880, PCM1883, Ye8 1B/O:8, WA-314, Ye9 2/O:9 and Ye3 4/O:3). GR- Gene Ruler™ – DNA ladder.

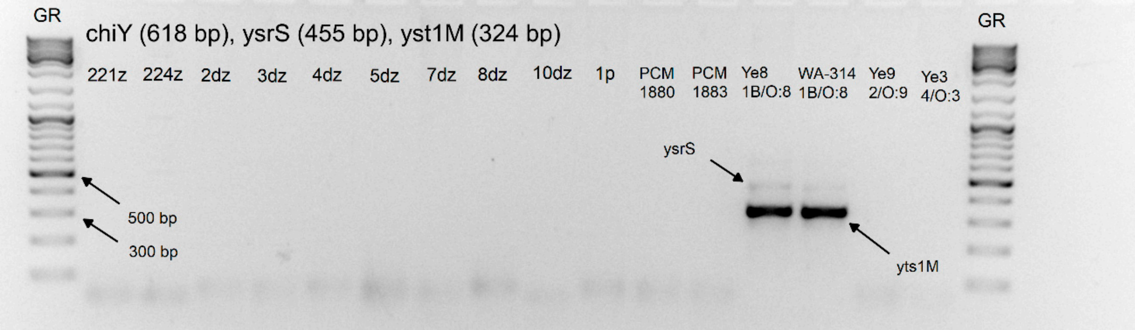

Figure S3. Gel electrophoresis after amplification of the *chiY*, *ysrS* and *yst1M* virulence genes of selected *Yersinia* sp. strains isolated from fattening pigs (221z-224z), wild boars (2dz-10dz), dogs (1p) and reference strains (PCM 1880, PCM 1883, Ye8 1B/O:8, WA-314, Ye9 2/O:9 and Ye3 4/O:3). GR- Gene Ruler™ – DNA ladder.

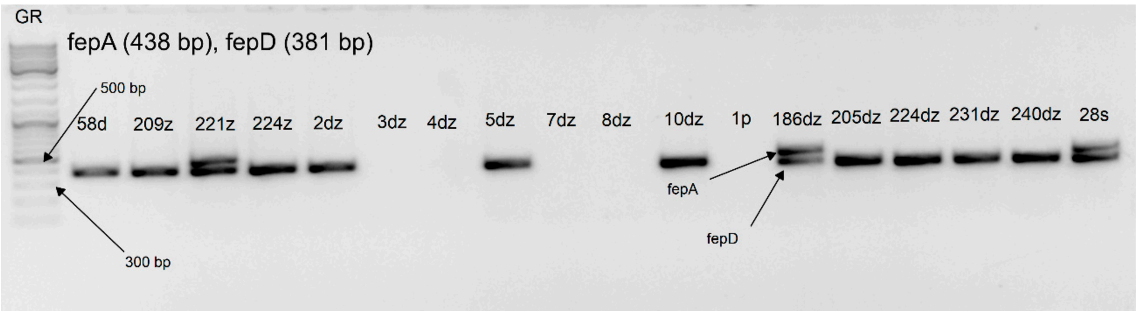

Figure S4. Gel electrophoresis after amplification of the *fepA* and *fepD* virulence genes of selected *Yersinia* sp. strains isolated from human faeces (58d), fattening pigs (209z-224z), wild boars (2dz-10dz, 186dz-240dz), dogs (1p) and roe deer (28s). GR- Gene Ruler™ – DNA ladder.

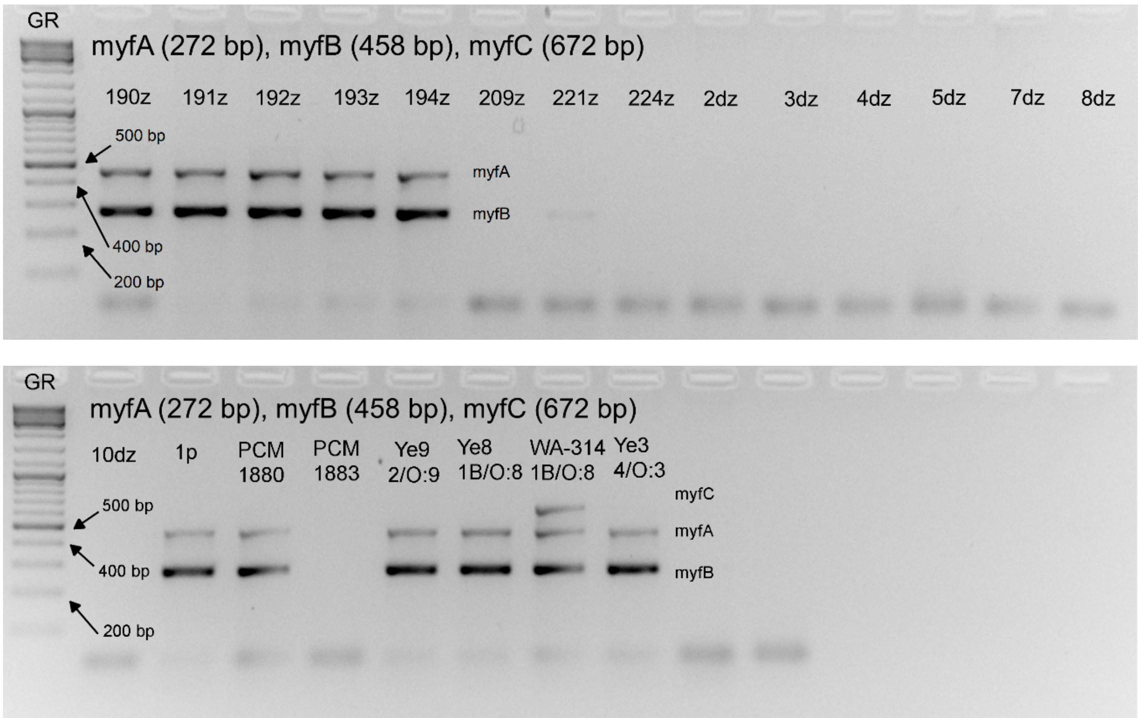

Figure S5. Gel electrophoresis after amplification of the *myfA*, *myfB* and *myfC* virulence genes of selected *Yersinia* sp. strains isolated from fattening pigs (190z-224z), wild boars (2dz-10dz), dogs (1p), and reference strains (PCM 1880, PCM 1883, Ye9 2/O:9, Ye8 1B/O:8, WA-314 1B/O:8 and Ye3 4/O:3). GR- Gene RulerTM – DNA ladder.

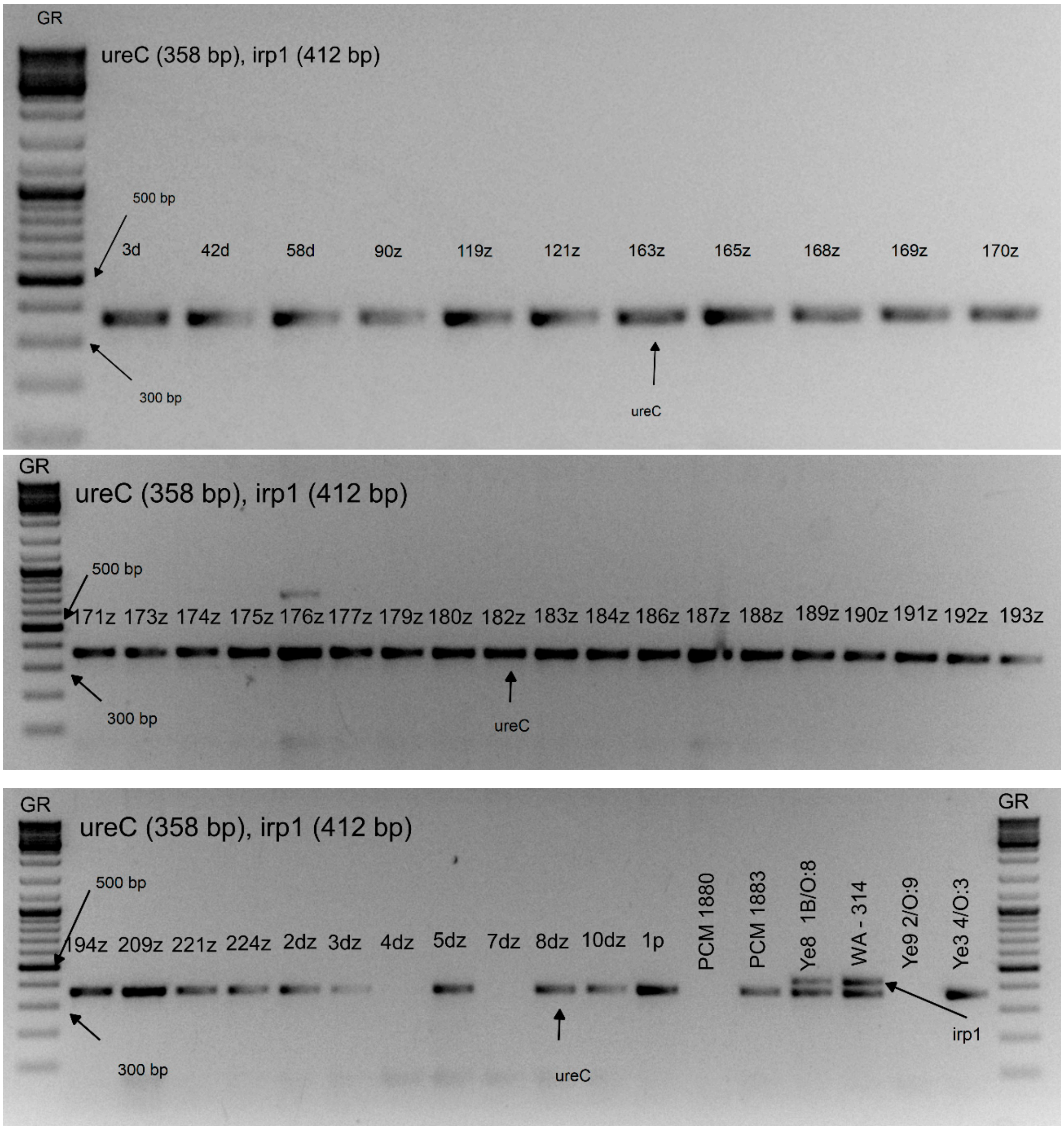

Figure S1. Gel electrophoresis after amplification of the *ureC* and *irp1* virulence genes of selected *Yersinia* sp. strains isolated from human faeces (3d-58d), fattening pigs (90z-224z), wild boars (2dz-10dz), dogs (1p) and reference strains (PCM 1880, PCM 1883, Ye8 1B/O:8, WA-314, Ye9 2/O:9 and Ye3 4/O:3). GR- Gene Ruler™ – DNA ladder.

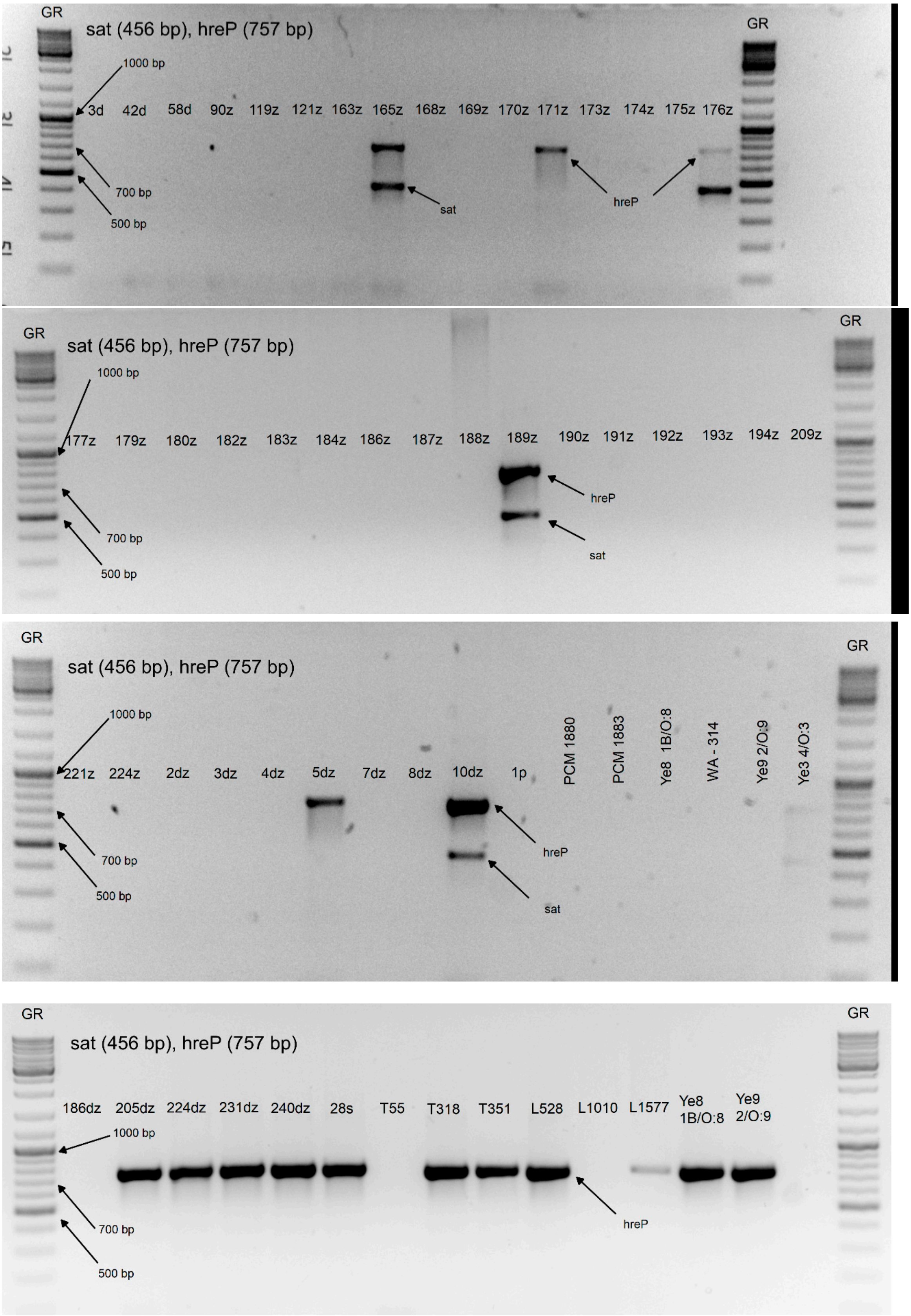

Figure S2. Gel electrophoresis after amplification of the *sat* and *hreP* virulence genes of selected *Yersinia* sp. strains isolated from human faeces (3d-58d), fattening pigs (90z-224z), wild boars (2dz-240dz), roe deer (28s), dogs (T318, T351, L528, 1p) and reference strains (PCM 1880, PCM 1883, Ye8 1B/O:8, WA-314, Ye9 2/O:9 and Ye3 4/O:3). Isolates T55, L1010, L1577 are not included in publication. GR- Gene Ruler™ – DNA ladder.

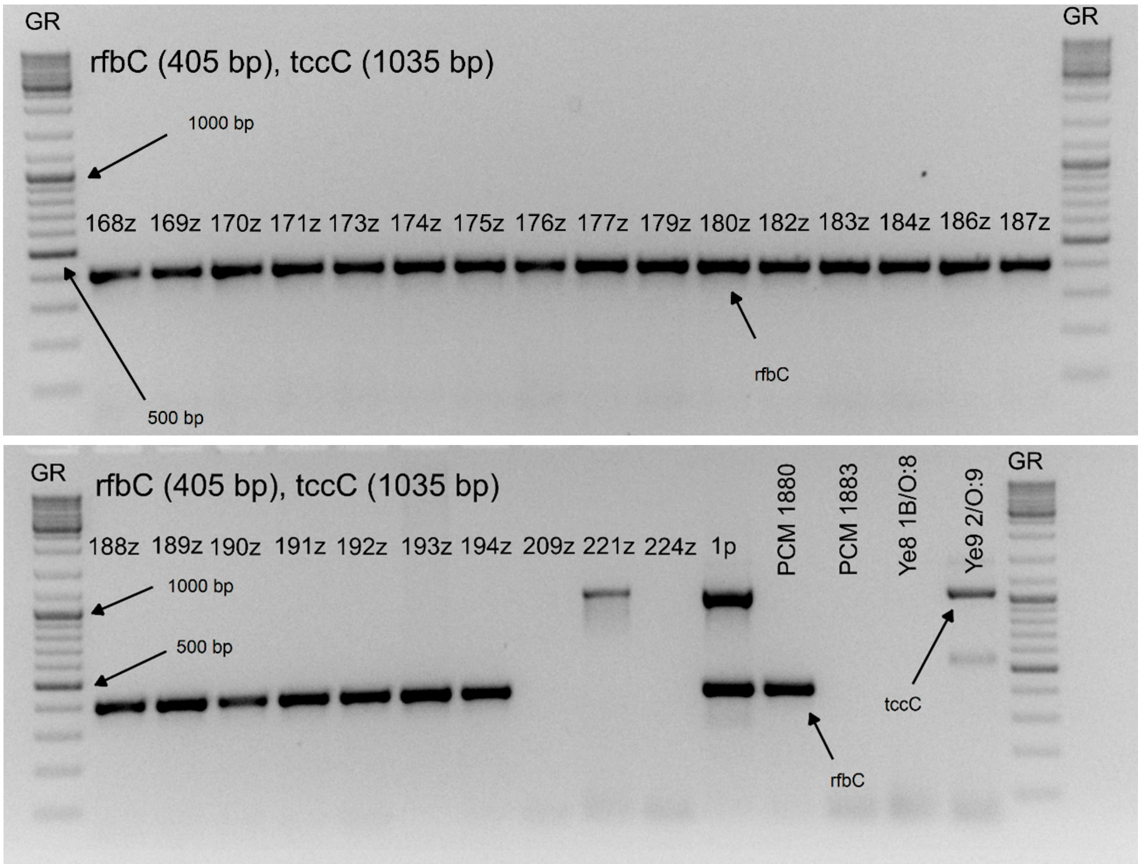

Figure S3. Gel electrophoresis after virulence genes *rfbC* and *tccC* amplification of selected *Yersinia* sp. strains isolated from fattening pigs (168z-224z), dogs (1p) and reference strains (PCM 1880, PCM 1883, Ye8 1B/O:8 and Ye9 2/O:9). GR- Gene Ruler™ – DNA ladder.

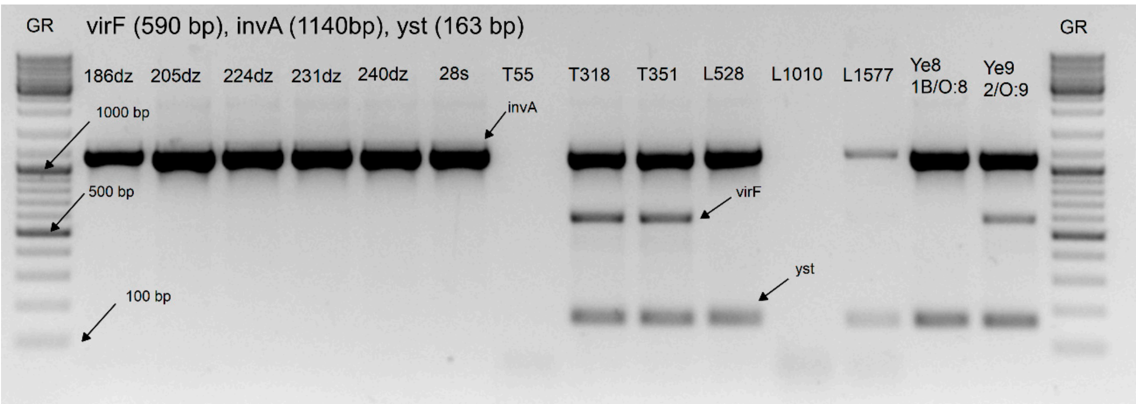

Figure S4. Gel electrophoresis after virulence genes *virF*, *invA*, *yst* amplification of selected *Yersinia* sp. strains isolated from wild boars (186dz-240dz), dogs (T318, T351, L528), roe deer (28s) and reference strains (Ye8 1B/O:8 and Ye9 2/O:9). GR- Gene Ruler™ – DNA ladder.

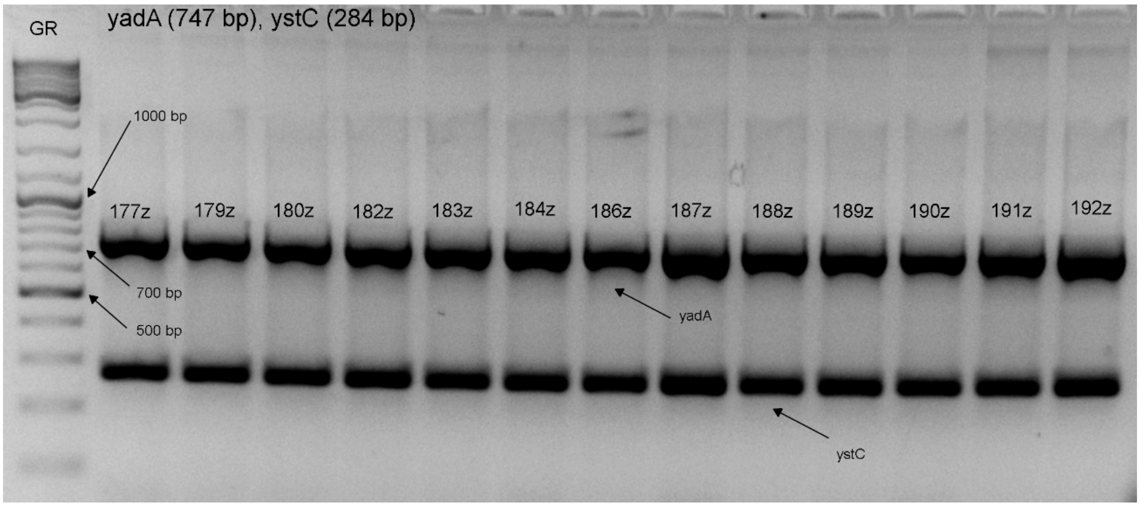

Figure S5. Gel electrophoresis after virulence genes *yadA*, *ystC* amplification of selected *Yersinia* sp. strains isolated from fattening pigs (177z-192z). GR- Gene Ruler™ – DNA ladder.

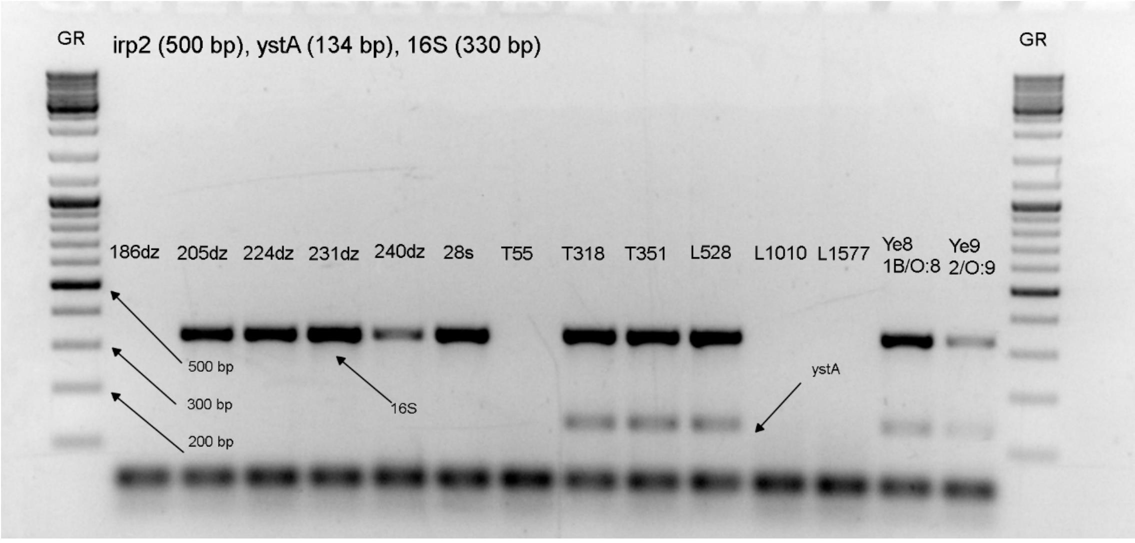

Figure S6. Gel electrophoresis after amplification of the *irp2*, *ystA*, 16S (rDNA) virulence genes of selected *Yersinia* sp. strains isolated from wild boars (186dz-240dz), dogs (T318, T351, L528), roe deer (28s) and reference strains (Ye8 1B/O:8 and Ye9 2/O:9). Isolates T55, L1010, L1577 are not included in publication. GR- Gene Ruler™ – DNA ladder.

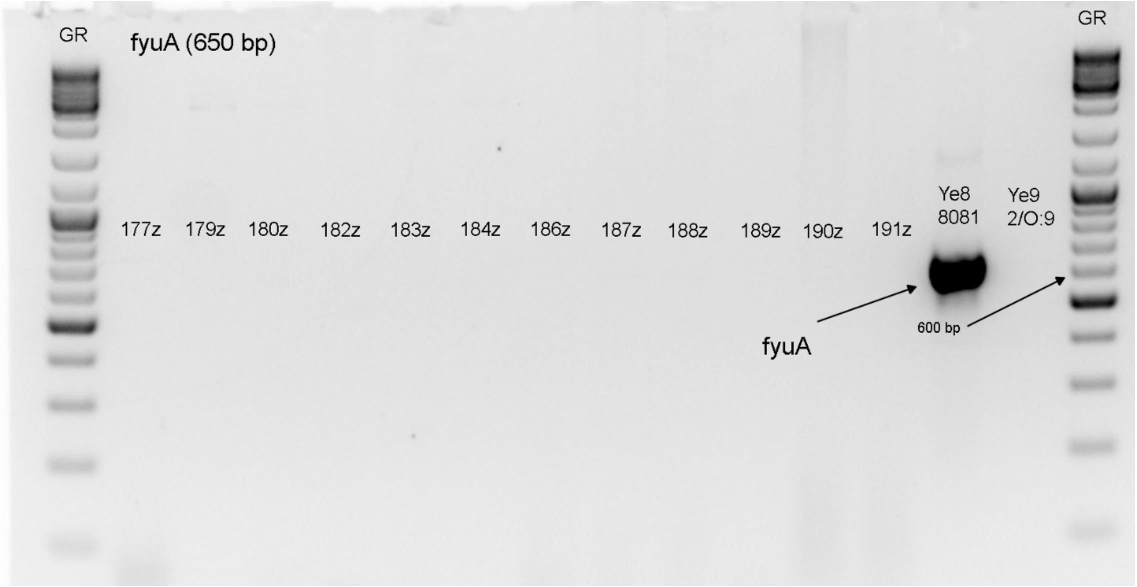

Figure S7. Gel electrophoresis after virulence genes *fyuA* amplification of selected *Yersinia* sp. strains isolated fattening pigs (177z-191z). and reference strains (Ye8 1B/O:8 and Ye9 2/O:9). GR- Gene Ruler™ – DNA ladder.

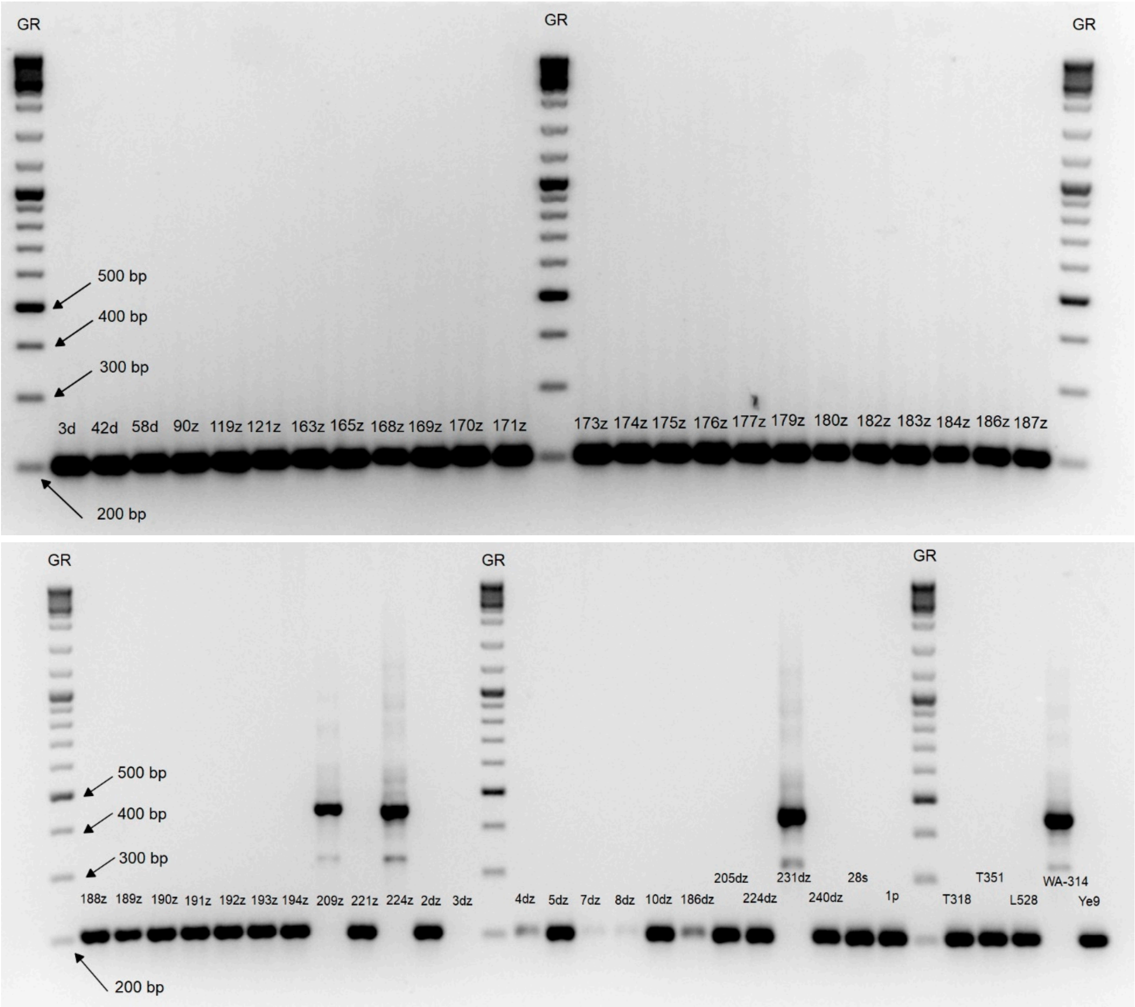

Figure S8. Polymorphism of tandem repeats within the VNTR A of tested *Yersinia* sp. strains isolated from human faeces (3d-58d), fattening pigs (90z-224z), wild boars (2dz-240dz), roe deer (28s), dogs (1p, T318, T351, L528) and reference strains (WA-314 1B/O:8, Ye9 2/O:9). GR- Gene RulerTM – DNA ladder. Exemple: Number of A repeats in $Y.\,enterocolitica$ 3d isolate $= \frac{200-177}{124} \sim 0$.

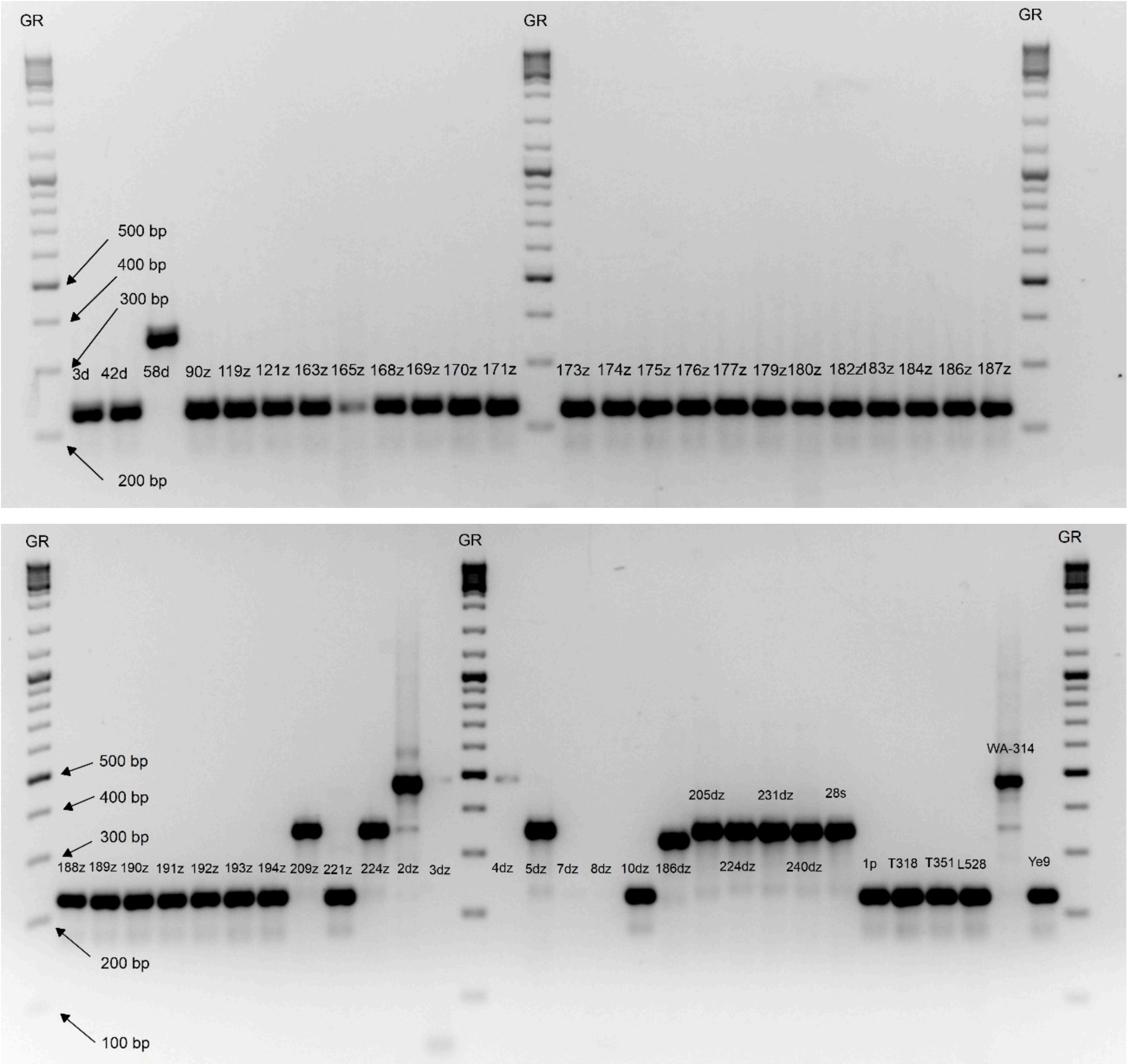

Figure S9. Polymorphism of tandem repeats within the VNTR B of tested *Yersinia* sp. strains isolated from human faeces (3d-58d), fattening pigs (90z-224z), wild boars (2dz-240dz), roe deer (28s), dogs (1p, T318, T351, L528) and reference strains (WA-314 1B/O:8, Ye9 2/O:9). GR- Gene RulerTM – DNA ladder. Example: Number of B repeats in $Y. enterocolitica$ 58d isolate $= \frac{350-167}{128} \sim 2$.

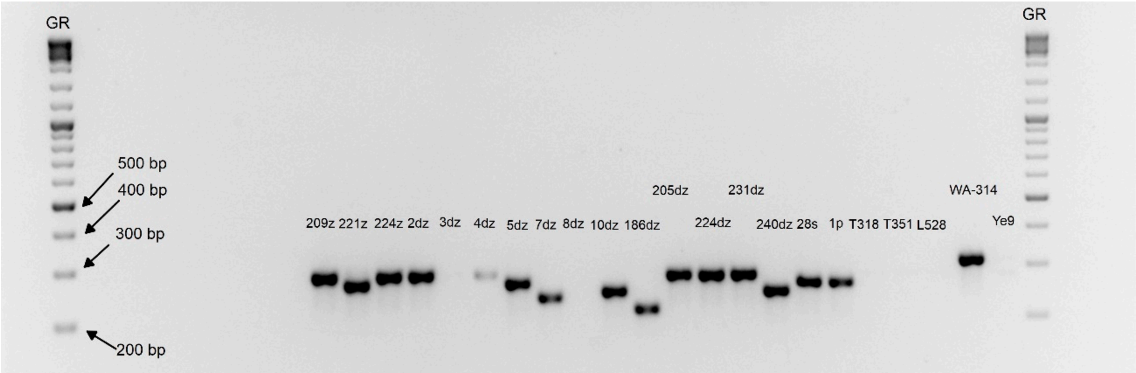

Figure S15. Polymorphism of tandem repeats within the VNTR C of tested *Yersinia* sp. strains isolated from fattening pigs (209z-224z), wild boars (2dz-240dz), roe deer (28s), dogs (1p, T318, T351, L528) and the reference strains (WA-314 1B/O:8, Ye9 2/O:9). The remaining group of strains did not possess the tandem repeat of C region within the genome. GR- Gene Ruler™ – DNA ladder. Example: Number of C repeats in $Y. enterocolitica$ 2dz isolate $= \frac{280-203}{34} \sim 3$.

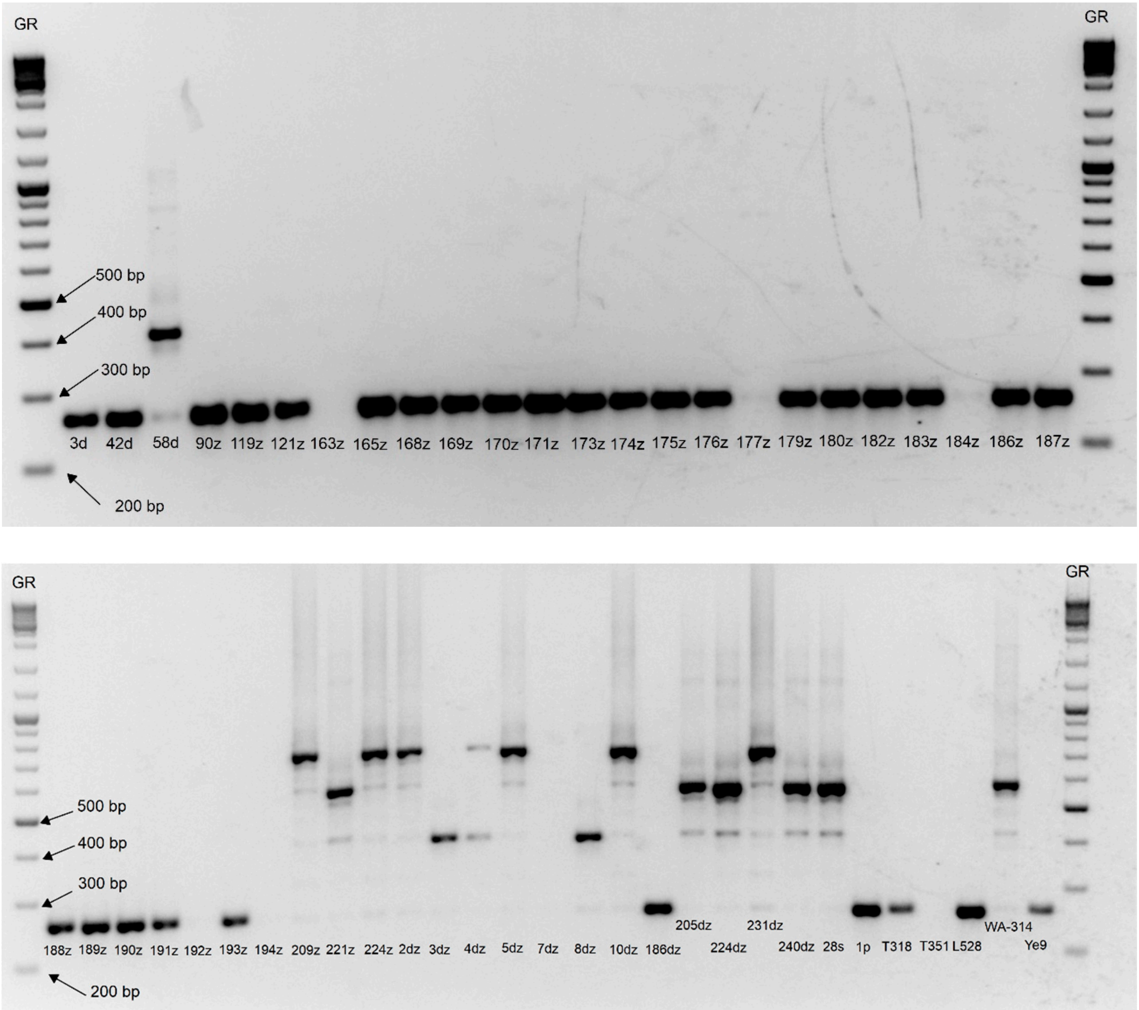

Figure S16. Polymorphism of tandem repeats within the VNTR D of tested *Yersinia* sp. strains isolated from human faeces (3d-58d), fattening pigs (90z-224z), wild boars (2dz-240dz), roe deer (28s), dogs (1p, T318, T351, L528) and reference strains (WA-314 1B/O:8, Ye9 2/O:9). GR- Gene RulerTM – DNA ladder. Example: Number of D repeats in $Y. enterocolitica$ 209z isolate $= \frac{750-204}{160} \sim 4$.

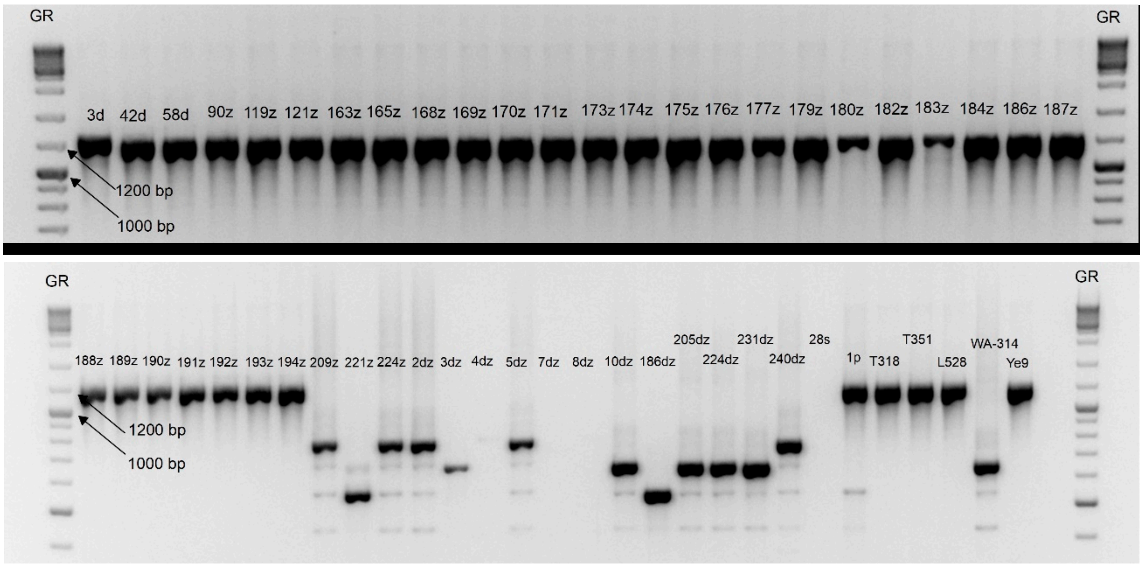

Figure S17. Polymorphism of tandem repeats within the VNTR E of tested *Yersinia* sp. strains isolated from human faeces (3d-58d), fattening pigs (90z-224z), wild boars (2dz-240dz), (28s), dogs (1p, T318, T351, L528) and reference strains (WA-314 1B/O:8, Ye9 2/O:9). GR- Gene RulerTM – DNA ladder. Example: Number of E repeats in $Y. enterocolitica$ 187z isolate $= \frac{1200-260}{114} \sim 9$.

Table S1. Primers used in PCR detection of virulence-associated genes in *Yersinia* sp. strains.

Gene	Gene product/ function	Primers sequence (5'→3')	Primers concentration [μM]	Amplicon size [bp]	PCR conditions [°C, s]			
					Denaturation	Annealing	Extension	Reference
yadA	Marker for the presence of virulence plasmid pYV. Complement resistance	TAAGATCAGTGTCTCTGCGGCA<br>TAGTTATTTGCGATCCCTAGCAC	0.15	747	94, 60	58, 90	72, 90	[60]
virF	Transcriptional activator	CATGGCAGAACAGCAGTCAG<br>ACTCATCTTACCATTAAGAAG	0.15	590	94, 60	58, 90	72, 90	[60]
ail	Adhesive-invasive protein of pathogenic biotypes of *Y. enterocolitica*	TAGTTCTCTAATAGCCTGTTTATC<br>ACTATCTGAGATGATTAGAATCG	0.15	531	94, 45	50, 60	72, 45	[38]
yst	Enterotoxin	AATGCTGTCTTCATTTGGAGC<br>GCAACATACATCACAGCAATC	0.15	163	94, 60	58, 90	72, 90	[64]
ystA	Enterotoxin YstIA of pathogenic biotypes (1B, 2-5)	GTCTTCATTTGGAGGATTCGGC<br>AATCACTACTGACTTCGGCTGG	1.25	134	94, 45	54, 45	72, 45	[38]
ystB	Enterotoxin YstIB of nonpathogenic biotype 1A, that control the production of heat stable	TGTCAGCATTTATTCTCAACT<br>GCCGATAATGTATCATCAAG	0.4	180	94, 45	50, 60	72, 45	[38], [39]

	enterotoxins							
ystC	Enterotoxin YstIC of nonpathogenic biotype 1A that control the production of heat stable enterotoxins	TCGACAAGTGAGTGACGGAG<br>CCCTTACTCGCGACGAAATA	0.2	284	94, 60	58, 90	72, 90	[38], [39]
ysrS	Chromosomal secretion III system termed Ysa	GCTCCTCATTACATAAATCGG<br>ATTCTCTCGTACAGATAGCG	0.2	618	94, 45	58, 45	72, 45	[65]
myfA	The basic component of the Myf antigen involved in the colonization of the intestine	CAGATACACCTGCCTTCCATCT<br>CTCGACATATTCCTCAACACGC	0.2	272	94, 45	58, 45	72, 45	[65]
myfB	Specific periplasmic protein responsible for the proper architecture of the Myf antigen	AAGTTTTCAGTGAGGACTGG<br>CTGTTGTCCATTACGGTGCC	0.2	458	94, 45	58, 45	72, 45	[65]
myfC	Outer membrane protein responsible for the proper architecture of the Myf antigen	CTGAATCTCAATTGGTCGCG<br>GTAAATCGGTAGTTTCCAGC	0.2	672	94, 45	58, 45	72, 45	[65]

irp1	Yersiniabactin synthetase HMWP, characteristic of the epidemic strain 1B/O:8	GTACAGACCGCCTGCTCCAGTT TGTAACCTACCTGCCTGTCGTC	0.15	412	94, 60	58, 90	72, 90	[65]
irp2	Yersiniabactin HMWP2, characteristic of the epidemic strain 1B/O:8	CTCCGCAGAACAGGTAGCCGA CGACATACTCAATCTGTCCGG	1.25	500	94, 45	58, 45	72, 45	[65]
fyuA	The receptor responsible for taking siderophore with Fe^{3+}	CTACGACATGCCGACAATGCC TGCTTCCCGCGCCATAACGTG	0.2	650	94, 30	59, 45	72, 45	[65]
Yts1M	Component of the *Yersinia* chromosomal type II secretion systems termed Yts1	ACACAAAACCTACACAGCGC CAACGTGGTGATATGAACCC	0.2	324	94, 45	58, 45	72, 45	[65]
chiY	Chromosomal gene coding secretion substrat in Yts1 type II secretion system of *Y. enterocolitica*, putative chitin-binding protein	CGATTCATTAGATCTGACGC TCGAAATGAATAGCCAGTGC	0.2	618	94, 45	58, 45	72, 45	[65]

inv	Invasin	TGCCTTGGTATGACTCTGCTTCA<br>AGCGCACCATTACTGGTGGTTAT	0.15	1140	94, 60	58, 90	72, 90	[9]
tccC	Insecticidal toxin complex-like protein	GGGCAAAAAATGCGTGAAGAGAG<br>TTTACCGGAATAACGCACAGTTTTA	0.24	1035	94, 30	59, 60	72, 60	[9]
hreP	Subtilisin/kexin-like protease	GCCGCTATGGTGCCTCTGGTGTG<br>CCCGCATTGACTCGCCCGTATC	0.24	757	94, 45	69, 45	72, 45	[9]
fepA	Enterochelin receptor protein	TACGCCAAAATACCTTACGAT<br>TGTAAATACACCCCCACCTGA	0.24	438	94, 30	54, 60	72, 60	[9]
fepD	Enterochelin ABC transporter	GTGTGATTGCCTTACTATTG<br>CGGTCATCCTTTTATTACGG	0.24	381	94, 30	54, 60	72, 60	[9]
sat	Streptogramin acetyltransferase	CCGATGGTGGGGTTTTCTCAAG<br>GGGATTACCGCCGACCACACTA	0.24	456	94, 45	69, 45	72, 45	[9]
blaA	Class A constitutive broad spectrum penicillinase	AAATGCGCTACCGGCTTCAG<br>AGTGGTGGTATCACGTGGGT	0.1	439	95, 30	56, 30	72, 90	[66]
blaB	Class C inducible cephalosporinase (AmpC)	CCCACTTTATACCTTGGCACAAA<br>GAACATATCTCCTGCCTGGGAAAT	0.1	827	95, 30	56, 30	72, 90	[66]
rfbC	Marker characteristic for *Y. enterocolitica*	CGCATCTGGGACACTAATTCG<br>CCACGAATTCCATCAAAACCACC	0.24	405	94, 30	59, 60	72, 60	[67]

	serotype O:3							
ureC	Urease	CTGCGTGGATATGGTGAAGAGT<br>CATTAGAGAGCGCATGGTAAGC	0.15	358	94, 60	58, 90	72, 90	[18]
ymoA	*Y. enterocolitica chromosomal gene modulating the expression of virulence functions,* negative regulator of genes *yop, virF* and *inv*	GACTTTTCTCAGGGGAATAC<br>GCTCAACGTTGTGTGTCT	0.15	330	94, 45	50, 60	72, 45	[39]
